# Mobility and cognition are associated with wellbeing and health related quality of life among older adults: a cross-sectional analysis of the Vancouver Falls Prevention Cohort

**DOI:** 10.1186/s12877-015-0076-2

**Published:** 2015-07-05

**Authors:** Jennifer C. Davis, Stirling Bryan, Linda C. Li, John R. Best, Chun Liang Hsu, Caitlin Gomez, Kelly A. Vertes, Teresa Liu-Ambrose

**Affiliations:** Centre for Clinical Epidemiology and Evaluation, 828 West 10th Avenue, Vancouver, Canada; Vancouver Coastal Health Research Institute (VCHRI), Vancouver, BC V6T 2B5 Canada; Department of Physical Therapy, 2177 Wesbrook Mall, Vancouver, Canada; University of British Columbia, Vancouver, BC V6T 2B5 Canada; Arthritis Research Centre of Canada, 5591 No. 3 Road, Richmond, BC V6X 2C7 Canada; Aging, Mobility, and Cognitive Neuroscience Lab, 2211 Wesbrook Mall, Vancouver, Canada; Djavad Mowafaghian Centre for Brain Health, University of British Columbia & VCHRI, 2215 Wesbrook Mall, Vancouver, British Columbia V6T 1Z3 Canada; Center for Hip Health and Mobility, 311-2647 Willow Street, Vancouver, BC V5Z 1 M9 Canada

**Keywords:** Mobility, Cognition, Quality of life, Wellbeing

## Abstract

**Background:**

Ascertaining individuals’ quality of life and wellbeing is essential in public health and clinical research. The impact of these two pressing geriatric syndromes – impaired mobility and cognitive function -- on wellbeing and quality of life is not well examined. Hence, our objective was to identify key clinically relevant outcome measures of mobility and cognitive function that explain variation in wellbeing and health related quality of life (HRQoL) among community dwelling older adults.

**Methods:**

We conducted a cross-sectional analysis of 229 participants presenting to the Vancouver Falls Prevention Clinic from June 2010 through October 2013. The linear regression models included two dependent variables: the ICECAP-O assessing wellbeing and the EQ-5D-3L assessing HRQoL. Key independent variables included the Short Performance Physical Battery (SPPB) and the Montreal Cognitive Assessment (MoCA). Covariates included Functional Comorbidity Index (FCI), sex and age. In the two multiple linear regression models, age was statistically controlled. Other covariates (i.e., sex and FCI) were included based on statistical significance (i.e., *p* < 0.05).

**Results:**

The SPPB was significantly associated with HRQoL and with wellbeing after adjusting for known covariates (*p* < 0.05, Unstandardized ß (Standard Error) 0.023 (0.006) for HRQoL and 0.016 (0.003) for wellbeing). The MoCA was significantly associated with wellbeing after adjusting for known covariates (*p* = 0.006), Unstandardized ß (Standard Error) 0.005 (0.002) but not with health related quality of life (*p* > 0.05).

**Conclusion:**

We found that a measure of mobility and balance was associated with HRQoL and wellbeing. However, cognitive function was associated with wellbeing only. This study highlights the potential importance of considering wellbeing as an outcome measure if interventions are intended to have a broader impact than health alone.

## Background

Ascertaining individuals’ quality of life is a critically relevant activity for public health decision making and clinical research [29] and should be considered a priority. The outcomes of health care interventions are likely to have impact that extend broadly to quality of life outcomes [9]. For example, older adults who are able to maintain their mobility and overall functional independence are likely to feel more secure and a better general sense of wellbeing. Such feelings may not be reflected fully by ascertaining health related quality of life alone (HRQoL) as compared with quality of life/wellbeing. As such, examining quality of life more broadly may be an important supplement to accurately value the impact of various interventions aimed at combatting cognitive decline and mobility impairments among older adults.

Wellbeing can be assessed using the ICECAP-O index of capability, a preference-based outcome measure. Preference-based outcome measures are distinct from other health or wellbeing status instruments because they provide insight into individuals within society’s valuations of specific states of health or wellbeing status. The ICECAP-O was developed to provide a broader assessment of gains or losses that extend beyond health alone – wellbeing (ie., quality of life more broadly) [5, 31]. It is described by its developers as a measure of wellbeing and capability, conceptually linked to Sen's capability approach which defines wellbeing in terms of what individuals are able to do, not what individuals actually do [5, 6, 32, 33]. Specifically, this approach is based on assessing an individuals capability to achieve valued functionings [23]. Capabilities reflect an individual’s ability to perform specific tasks. Sen emphasizes that an individual’s capabilities are most useful in assessing impact [33].

HRQoL is frequently ascertained using the EQ-5D [24]. The EQ-5D three level (3L) (EQ-5D-3L) version captures 243 health states [24] and assesses an individual’s HRQoL according to the following attributes: mobility, self-care, usual activities, pain and, anxiety or depression. The EQ-5D is the most widely used generic instrument that uses a utility-based scoring approach, yielding a single summary score (i.e., Health State Utility Value (HSUV)) on a common scale to facilitate comparison across different health conditions and patient populations [24]. The HSUV is anchored at zero – a health state equivalent to death and 1.0 – a state defined as “full health”. HSUVs less than zero define health states worse than death. The EQ-5D is one example of a tool that is used to attach a metric to measure ‘health’. HSUVs are a highly relevant and important outcome in both clinical research and clinical practice. Yet, they may not fully capture quality of life outcomes more broadly. As such, it is possible that HSUVs may underestimate potential benefits of health care or public health interventions.

Impaired cognitive and mobility critically impact older adult's HRQoL and wellbeing [2, 26]. Impaired mobility is a major concern for older adults and is associated with greater risk for disability, institutionalization, and death [30]. Cognitive impairments and mobility issues often co-exist and their temporal relationship appears to be bi-directional. Impaired mobility is becoming recognized as a neurological biomarker of dementia during preclinical stages [4]. Current evidence also suggests cognitive decline and mobility share common underlying pathophysiology (i.e., vascular pathology and inflammation) [7, 13]. Specifically, the Health, Aging and Body Composition Study [1] demonstrated that baseline lower executive functions predicted subsequent decline in gait speed. Recently, rates of decrease in gait speed were shown to be significantly different between older adults who developed MCI and those who did not [3]. Given that both impaired cognitive function and impaired mobility contribute to loss of functional independence which is associated with reduced quality of life, greater risk for institutionalization, and increased mortality – there is a critical need to further investigate the specific contribution of cognitive functioning and mobility to HRQoL and wellbeing. Understanding key determinants of HRQoL and wellbeing will help inform future intervention strategies aimed at combatting cognitive and functional decline and thus striving to maintain or improve individual’s HRQoL and wellbeing.

Hence, the objective of our study was to determine and compare key factors relating to mobility and cognitive function that explain significant variation in HRQoL and capability/wellbeing among community dwelling older adults.

## Methods

### Study design

We conducted a cross-sectional analysis of a cohort of 229 participants (complete case analysis) who presented to the Vancouver Falls Prevention Clinic from June 2010 through October 2013 for a baseline assessment.

Ethical approval was obtained from the Vancouver Coastal Health Research Institute and the University of British Columbia’s Clinical Research Ethics Board (H09-02370). All participants provided written informed consent.

### Participants

The sample consisted of women and men referred by their general practitioner or emergency department physician to the Vancouver Falls Prevention Clinic. Community dwelling women and men who lived in the lower mainland region of British Columbia were eligible for study entry if they:

- were adults ≥ 70 years of age referred by a medical professional to the Falls Prevention Clinic as a result of seeking medical attention for a non-syncopal fall in the previous 12 months;
- understood, spoke, and read English proficiently;
- had a Physiological Profile Assessment (PPA) [21] score of at least 1.0 SD above age-normative value or Timed Up and Go Test (TUG) [36] performance of greater than 15 seconds or one additional non-syncopal fall in the previous 12 months;
- were expected to live greater than 12 months (based on the geriatricians’ expert opinion);
- were able to walk 3 m with or without an assistive device; and
- were able to provide written informed consent.

We excluded those with a neurodegenerative disease (e.g., Parkinson’s disease) or dementia, patients who has a stroke in the past 12 months, those with clinically significant peripheral neuropathy or severe musculoskeletal or joint diseases, and anyone with a history indicative of carotid sinus sensitivity (i.e., syncopal falls).

### Vancouver falls prevention clinic measures

A comprehensive set of measurements relating to mobility and cognitive function that were collected are described below.

### Outcome measures

The primary outcomes of interest were wellbeing and HRQoL.

#### Wellbeing

We assessed wellbeing using the ICECAP-O [5, 6, 17]. The ICECAP-O is a five item multiple choice questionnaire that measures an individual’s wellbeing and quality of more broadly according to five attributes: attachment (love and friendship), security (thinking about the future without concern), role (doing things that make you feel valued), enjoyment (enjoyment and pleasure) and control (independence). Each domain has four possible response options. The ICECAP-O can be used to calculate a global capability index score on a zero to one scale where zero represents no capability and one represents full capability.

#### Health related quality of life

We assessed HRQoL using the EQ-5D three level version (3 L). The EQ-5D-3L is a short five item multiple choice questionnaire that measures an individual’s HRQoL and health status according to the following five domains: mobility, self-care, usual activities, pain and anxiety/depression [11]. Each domain has three possible response options indicating no problems, some problems or severe problems. The EQ-5D-3L health state utility values (HSUVs) at each time point are bounded from −0.54 to 1.00 where a score of less than zero is indicative of a health state worse than death. The HSUVs represent values that individuals within society assign -- values for specific health states such as having rheumatoid arthritis relative to perfect health -- these are UK societal values for given health states.

### Predictor variables

The Short Physical Performance Battery (SPPB) [15] was used to assess mobility and balance. For the Short Physical Performance Battery, participants were assessed on performances of standing balance, walking, and sit-to-stand. Each component is rated out of four points, for a maximum of 12 points; a score < 9/12 predicts subsequent disability [16].

### Executive functions

There is no unitary executive function – rather, there are distinct processes. Three key executive processes that are distinct processes include: 1) selective attention and conflict resolution (or response inhibition) ^31^; 2) set shifting; and 3) updating (or working memory). Executive functions will be assessed using the Montreal Cognitive Assessment (MoCA). The MoCA is a brief screening tool for MCI [27] with high sensitivity and specificity, was used to categorise participants as with, or without, possible MCI. It is a 30-point test covering eight cognitive domains: 1) attention and concentration; 2) executive functions; 3) memory; 4) language; 5) visuo-constructional skills; 6) conceptual thinking; 7) calculations; and 8) orientation. Scores below 26 are considered to be indicative of possible MCI. A bonus point is given to individual’s with less than 12 years of education. Information processing speed will be indexed using the Digit Symbol Substitution Test (DSST) ^35^. For this task, participants first present with a series of numbers (1 to 9) and their corresponding symbols. They are asked to draw the correct symbol for any digit - placed randomly in pre-defined series - in 60 s. A higher number of correct answers in this time period indicated a better executive functions and processing speed.

### Descriptive variables

Physiological falls risk was assessed using the short form of the Physiological Profile Assessment (PPA). The PPA is a valid [58, 59] and reliable [60] measure of falls risk. Based on a participant’s performance in five physiological domains – postural sway, reaction time, strength, proprioception, and vision – the PPA computes a falls risk score (standardized score) that has a 75 % predictive accuracy for falls in older people [20, 22]. A PPA Z-score of ≥ 0.60 indicates high physiological falls risk [10].

We assessed global cognition using the Mini Mental State Examination (MMSE). The MMSE is a widely used and well-known questionnaire used to screen for cognitive impairment (i.e., MMSE <24) [12]. It is scored on a 30-point scale with a median score of 28 for healthy community dwelling octogenarians with more than 12 years of education [12]. The MMSE may underestimate cognitive impairment for frontal system disorders because it has no items specifically addressing executive function [12].

Functional comorbidity index (FCI) was calculated to estimate the degree of comorbidity associated with physical functioning [14]. This scale’s score is the total number of comorbidities. We also collected information relating to living status (i.e., alone, with others or assisted living) and level of education.

### Statistical analysis

We analyzed all data using STATA version 10.1. We report descriptive data for all variables of interest for this cross-sectional analysis. For data that are normally distributed we report mean and standard deviation and frequencies (%) depending on the measure. The nature of the relationship between the continuous independent (SPPB, PPA, MoCA and MMSE) and dependent variables (ICECAP-O and EQ-5D-3L) of interest were examined using two-way scatter plots. Bivariate relationships between the independent variables and the two dependent variables of interest were ascertained using Pearson correlations. Linear regression models were constructed with the following two dependent variables: wellbeing (assessed using the ICECAP-O) and HRQoL (assessed using the EQ-5D-3L). Independent variables included the SPPB, PPA, MoCA and MMSE. Covariates investigated in the bivariate analysis included FCI, sex and age. In our two multiple linear regression models (i.e., using the two dependent variables: wellbeing and HRQoL), age was statistically controlled by forcing this variable into both regression models. Other covariates (i.e., sex and FCI) were kept in based on their statistical significance. Co-linearity of all variables was ascertained and for variables that were highly co-linear, the variable with the strongest bivariate association was included in the final regression model. We assessed the assumptions of normality of the residuals and heteroscedasticity. Lastly, we conducted exploratory domain specific comparisons of the ICECAP-O and the EQ-5D-3L with the SPPB. We used Spearman correlation coefficients for the specific domains of the EQ-5D (mobility, self-care, usual activities, pain and depression) & ICECAP-O (attachment, security, role, enjoyment and control) with the SPPB.

## Results

Two-hundred and twenty-nine participants are included in our analysis.

### Participants

Table 1 reports descriptive statistics for our variables of interest for this cohort. This cohort of community-dwelling older adults had a mean (SD) EQ-5D-3L HSUV of 0.78 (0.22) and a mean (SD) ICECAP-O of 0.82 (0.12). Participants were classified as having high falls risk with a mean PPA score of 1.6 ± 1.0. Further, the mean MoCA score was 22 ± 4.

**Table 1:** Characteristics of the Vancouver Falls Prevention cohort (*n* = 229)

Variables	Mean (SD) or Number (%)
EQ-5D-3L	0.785 (0.218)
ICECAP-0	0.819 (0.122)
Age (years)	82.4 (6.7)
Living status (*n* = 186)	
Lives alone	68 (36.6 %)
Lives with others	94 (50.5 %)
Assisted living	24 (12.9 %)
Education (*n* = 220) < Grade 9	18 (8.2 %)
Grades 9–13, no diploma	44 (20 %)
High school with diploma	44 (20 %)
Trades school	17 (7.8 %)
Some university	29 (13.2 %)
University	68 (30.0 %)
Sex (Male/Female)	79/150 (34.5 %/65.5 %)
FCI	2.5 (1.9)
SPPB^a^	7.2 (2.5)
PPA^b^	1.6 (1.0)
MMSE (max 30 pts)	26.7 (2.6)
MoCA (max 30 pts)	22.1 (4.5)
DSST	19.8 (7.6)

FCI: Functional Comorbidity Index

SPPB: Short Performance Physical Battery

PPA: Physiological Profile Assessment

MMSE: Mini-Mental State Examination

MoCA: Montreal Cognitive Assessment

DSST: Digit Symbol Substitution Test

^a^A SPPB score of < 9/12 predicts subsequent disability

^b^A PPA Z-score of ≥ 0.60 indicates high physiological falls risk

### Correlation coefficients

Table 2 reports the correlation coefficients between independent variables of interest and both health related quality of life (EQ-5D-3L) and wellbeing (ICECAP-O). The FCI (*p* < 0.01) and sex (*p* < 0.05) were significantly associated with health related quality of life. The strength of the correlation for sex was negligible and the strength of the correlation for FCI was weak. The SPPB (*p* < 0.01), PPA (*p* < 0.05), MoCA (*p* < 0.05) and DSST (*p* < 0.05) were significantly associated with wellbeing. The strength of the correlation was moderate for the SPPB, negligible for the PPA, weak for the MoCA and negligible for the DSST. The SPPB, was significantly associated with both health related quality of life and wellbeing (*p* < 0.01). Measures of executive functions (i.e., MoCA and DSST) were not significantly associated with health related quality of life. In contract, measures of executive functions were significantly associated with wellbeing.

**Table 2:** Correlation coefficient matrix (*n* = 229)

Variables	EQ-5D-3L	ICECAP-O
Age (years)	0.0933	−0.0904
Sex (Male/Female/Both)	−0.126*	−0.0473
FCI	−0.212**	−0.0805
SPPB	0.256**	0.353**
PPA	0.0792	−0.157*
MMSE (max 30 pts)	−0.124	0.0748
MoCA (max 30 pts)	−0.0841	0.236*
DSST	−0.0077	0.163*

*p < 0.05

**p < 0.001

FCI: Functional Comorbidity Index

SPPB: Short Performance Physical Battery

MoCA: Montreal Cognitive Assessment

DSST: Digit Symbol Substitution Test

### Multivariate linear regression

The SPPB was significantly associated with HRQoL and wellbeing after adjusting for (age, FCI and sex for HRQoL and age, sex and MoCA for wellbeing) (*p* < 0.05). The total variance accounted for by the final model for health related quality of life was 13 % and for wellbeing was 15 % (Table 3). The SPPB accounted for an additional 7 % of the total variance in the final model for health related quality of life. The SPPB accounted for an additional 10 % of the total variance in the final model for wellbeing. The MoCA accounted for an additional 3 % of the total variance in the final model for wellbeing.

**Table 3:** Multiple Linear Regression Summary examining the contribution of mobility and/or cognition function on health related quality of life and wellbeing (*n* = 229)

		EQ-5D-3 L	
Independent Variables	R^2^	Unstandardized ß	*P*-value
		(Standard Error)	
Model^a^	0.134		
Age		0.005 (0.002)	0.024*
FCI		−0.018 (0.007)	0.013*
Sex (Male/Female)		−0.06 (0.03)	0.045*
SPPB		0.023 (0.006)	0.000**
		ICECAP-O	
Model^b^	0.154		
Age		0.0006 (0.0012)	0.636
MoCA		0.005 (0.002)	0.006**
SPPB		0.016 (0.003)	0.000**

*p < 0.05

**p < 0.001

^a^Model 1: Additional variation explained by the SPPB = 8.4 %

^b^Model 2: Additional variation explained by the MoCA = 5.1 % and Model 2: Additional variation explained by the SPPB = 9.6 %

FCI: Functional Comorbidity Index

SPPB: Short Performance Physical Battery

MoCA: Montreal Cognitive Assessment

### Domain specific comparisons of the EQ-5D-3L and the ICECAP-O with the SPPB

Four of the five EQ-5D domains (mobility, self-care, usual activities and pain) were significantly associated with the SPPB (Table 4). Four of the five ICECAP-O domains (i.e., attention, role, enjoyment and control) were significantly associated with the SPPB.

**Table 4:** Spearman Correlation Coefficient Matrix Summary for a Measure of Balance and Mobility with Health Related Quality of Life and Wellbeing Domains

Instrument	Short Physical Performance Battery
EQ-5D Individual Domains	
Mobility	−0.2577*
Self-Care	−0.1295*
Usual activities	−0.1679*
Pain	−0.1400*
Depression	−0.1230
ICECAP-O Individual Domains	
Attachment	−0.1382*
Security	−0.1099
Role	−0.2048*
Enjoyment	−0.1749*
Control	−0.3615*

*p < 0.05

## Discussion

This study demonstrated that the MoCA, a measure of cognitive function and executive function, was significantly associated with wellbeing after accounting for known covariates and the SPPB. Of note, cognitive function was not significantly associated with HRQoL. Executive functions often decline substantially with aging [19]. Intact executive functioning is essential to the ability to carry out health-promoting behaviours [34], such as medication management, dietary and lifestyle changes, self-monitoring of responses, and follow-up with health care professionals. Wellbeing, assessed using the ICECAP-O, taps into an individual’s capability to achieve desired functionings (i.e., this can be thought of as an individual’s capacity to follow through with what they want to achieve). It is conceivable that an individual with higher executive functioning may be more competent in achieving their targets which may explain the significant association with wellbeing and not HRQoL.

The differential findings between the instruments assessing wellbeing and HRQoL highlight two important implications for future research. Given that both the EQ-5D and the ICECAP-O were largely developed for use in economic evaluations (i.e., a simultaneous evaluation of costs and effectiveness of intervention strategies), it is important to consider the consequences of our findings in this context. First, interventions aimed at combatting cognitive decline may often result in broader health benefits that may not be captured by assessing HRQoL alone [35]. Resultant economic evaluations of interventions may underestimate gains or losses in health status. Hence, it may be pertinent to consider measuring QoL more broadly. Second, cognition is not measured by directly by the EQ-5D or the ICECAP-O. The lack of association between the HRQoL and the MoCA may be the result of the EQ-5D not containing a domain that related to cognition – an issue previously debated in the literature [8]. The ICECAP-O also does not include a cognitive domain. However, by design the constructs and capabilities to achieve the desired functionings that comprise the ICECAP-O may better tap into aspects of cognitive function compared with the constructs of the EQ-5D. As such it is important to carefully consider the domains and constructs assessed when choosing an outcomes instrument to assess wellbeing.

We found that the SPPB, a valid and reliable measure of mobility and balance, explained significant variation in both HRQoL and wellbeing (Table 4). This observation may seem intuitive for the EQ-5D since one of the domains of the EQ-5D is mobility. One recent study demonstrated a correlation between lower EQ-5D scores and poor SPPB performance [18]. The ICECAP-O does not directly measure mobility. However, we found that the SPPB explained a larger amount of variation in the ICECAP-O score than the EQ-5D-3L score. Given that the ICECAP-O is a capability index – it is designed to ascertain an individual’s capability to achieve valued functionings [23]. Hence, it is highly conceivable that performance on the SPPB may be related to the domains of security (thinking about the future without concern), role (doing things that make you feel valued), enjoyment (things that make you feel valued). For example, it may be that having mobility allows you to do the things that you want to do and to do the things that makes you feel valued – the ICECAP-O is able to tap into individuals’ capabilities (i.e., their capability to achieve desired functionings).

We observed a significant association between sex and HRQoL. A significant sex effect was not detected for wellbeing. Previously, women previously reported not being content with their HRQoL even with normal physical function [28]. Further, one study demonstrated that women have poor mobility compared with men and report being most affected by their musculoskeletal status and depressive symptoms [25]. These are two symptoms that would be likely captured more directly by the EQ-5D domains of mobility, usual activities, pain and anxiety/depression.

We also noted that age explained a significant amount of variation in wellbeing but not in HRQOL. One explanation for this observation is that the ICECAP-O was designed specifically for older adults and may be more sensitive to detecting age related changes. The EQ-5D was designed for a general population of adults and thus may be less responsive among targeted populations such as older adults.

Participants included in this study were referred by health care providers to the study based on perceived fall risk and specifically sustaining a fall in the past 12 months. As such, the results of this study may not be generalizable to other low risk populations. On the other hand, this is an at-risk population for which findings are highly relevant for future targeted intervention. This cross-sectional analysis does not allow us to ascertain the temporal relationship between mobility and cognition in relation to HRQoL and wellbeing. This analysis was based on a complete case analysis. We chose not to report the imputed dataset here because the findings of the imputed data set concurred with the complete case analysis. Further, this study did not explore any type of mediation analyses. It is possible that risk of falls or falls self-efficacy could mediate the relationship between mobility or cognition and HRQoL or wellbeing. The next logical step is to conduct a longitudinal analysis ascertaining the key predictors and mediators of change in wellbeing and change in HRQoL over time. This will help us tailor and target future intervention strategies most effectively.

## Conclusions

This study highlights that both mobility and cognitive function are associated with HRQoL and wellbeing. Specifically, this study provides preliminary evidence that the ICECAP-O taps into important aspects of cognition – executive functions and the EQ-5D does not. As such, this study provides a platform for future longitudinal studies and intervention studies to 1) examine temporal relationships and mediating factors of mobility and cognition with HRQoL and wellbeing, 2) explore the use of appropriate instruments based on the intended impact of the intervention and 3) target mobility and cognition to improve wellbeing and slow age related declines.
